# Editorial: Omics technologies in livestock improvement: From selection to breeding decisions

**DOI:** 10.3389/fgene.2022.1113417

**Published:** 2023-01-04

**Authors:** Syed Mudasir Ahmad, Marcos De Donato, Basharat Ahmad Bhat, Abdoulaye Banire Diallo, Sunday O. Peters

**Affiliations:** ^1^ Division of Animal Biotechnology, Faculty of Veterinary Sciences and Animal Husbandry, SKUAST, Kashmir, India; ^2^ Escuela de Ingeniería y Ciencias, Instituto de Tecnología y Educación Superior de Monterrey (ITESM), Monterrey, Mexico; ^3^ The Center for Aquaculture Technologies, San Diego, CA, United States; ^4^ Departments of Surgical Sciences, University of Otago, Dunedin, New Zealand; ^5^ Department of Computer Science, Université du Québec à Montréal, Montreal, QC, Canada; ^6^ Department of Animal Science, Berry College, Mount Berry, GA, United States

**Keywords:** livestock, epigenomics, transcriptomics, GWAS, polymorphism, RNA sequencing

Livestock rearing is the main component of global food production systems and contributes to nearly half of global agricultural production. The emerging demand for animal products is considered a food revolution in developing countries. Although livestock farming is an essential component of the global economy but faces an immense challenge to meet out the increased demand of the exploding human population and other environmental factors. As a result, understanding animal health and production is becoming mandatory. Advances in genetic and genomic technologies have played a key role in improving animal welfare and productivity for decades. Genetic development in livestock, both in terms of productivity and other functional trait complexes associated with health and animal welfare, has been greatly aided by genomic selection, which is regarded as a success story. Genomic breeding programmes offer the potential to improve cattle productivity through the utilization of molecular genetics, the detection of markers and chromosomal areas that contain quantitative trait loci, and genome mapping technology. Present breeding strategies not only account for phenotypic variance in traits but also other factors like epigenetics. Epigenetic mechanisms (DNA methylation, RNA methylation, histone modifications, chromatin remodelling, and non-coding RNA regulation) have been strongly found to influence livestock traits like growth, development, and other phenotypic effects. Technologies related to omics are developing more frequently in the area of animal production. Other omics topics like phosphoproteomics, peptidomics, or lipidomics are presently used in livestock production and disease management in addition to omics methodologies such as transcriptomics, genomics, proteomics, and metabolomics. Animal breeding systems already in place now have additional dimensions due to the introduction of revolutionary ideas like animal cloning and genetic engineering. It provides a faster way to increase the frequency of desirable alleles in an animal population using genetically modified animals than traditional breeding procedures. With this background, the aim of the current research topic was to collect research data on the role of omics technologies in livestock improvement from selection to breeding decisions.

The special edition of Frontiers in Genetics i.e., “Omics Technologies in Livestock Improvement: From Selection to Breeding Decisions” published 26 original research articles, 2 review papers, and 01 methodology paper. All 29 articles were focused mainly on understanding the complex interplay of genes in mediating important traits, elucidating livestock genome/transcriptome, and improving livestock yield, nutrition values, and animal welfare.

Omics technologies are effective tools, especially when used in conjunction with advanced molecular and breeding methods. Genomic research in particular has the potential to improve the precision and efficacy of traditional breeding and advanced breeding approaches by enhancing consistency and predictability. Indeed, the information provided by genomics and other omics-technologies like epigenomics and proteomics is crucial for understanding genes and their function. Additionally, summarized the role of omics technologies in improving livestock (Chakraborty et al.).

Male infertility still poses a significant problem for animals, despite significant increases in reproductive efficiency brought about by artificial insemination procedures. Recent research indicates that the spermatozoa of fertile and infertile males have different populations of RNA. Studies have also shown that spermatozoal RNA (spRNA) is essential for spermatogenesis, fertilisation, and the early stages of embryonic development. Updated various spRNA species in livestock animals, including protein-coding and non-coding RNAs and their possible impact on quality of sperms, especially motility, freezability, and fertility (Sahoo et al.). The regulatory structure of circRNA in testis development and spermatogenesis in cattle bulls was evaluated using RNA sequencing by Khan et al., in immature and mature Wandong cattle bull testes. In calf and bull testes, 579 of the 17,013 circRNAs were elevated and 103 were downregulated. Bull spermatogenesis and the genes ATM, GSK3B, CCNA1, KMT2C, NSD2, KMT2E, QKI, SUCLG2, HOMER1, and SNAP91 were discovered to be strongly correlated. Through genetic selection, this information may aid in enhancing bulls’ reproductive productivity (Khan et al.).

Deep RNA sequencing was utilised to discover probable single nucleotide polymorphisms (SNP) in mammary epithelial cells from two different cow breeds (Jersey and Kashmiri) in order to study the alterations in coding regions that affect milk output differences. Ahmad et al., work seeks to find high-impact SNPs in Jersey and Kashmiri cows in order to uncover the main pathways controlling milk production features in each breed using RNA-Seq data (Ahmad et al.). There were discovered to be 684 (464 SNPs and 220 INDELs) and 607 (442 SNPs and 169 INDELs) high-impact variations unique to Kashmir and Jersey cattle, respectively. The RNA sequencing-based SNP research revealed a considerable difference between Kashmiri and Jersey cattle in terms of milk production attributes. The high-impact SNP variations in Kashmiri cattle contributed to adaptive immunity and tolerance to infectious diseases of the mammary gland. Contrarily, in Jersey cattle, enhanced pathways were mostly implicated in production and lactation maintenance. These findings offer information on breed-specific genetic diversity that can be applied to the genomic selection of animals. There are many studies like RNA sequencing leads and high-throughput genomic DNA data, which have identified the genes related to lipid storage in broilers. Using RNA-Seq and microarray data techniques, Farzad Ghafouri et al. identified and categorised candidate genes and miRNAs involved in lipid metabolism. Based on the amount of abdominal fat present, two broiler groups were selected: high and low. In the analysis, 34 genes and 19 miRNAs were detected as common. A total of seven genes were revealed as common, three being most important *viz.*, REBF1, SREBF2, SCD, and FASN. These have a significant impact on fat metabolism, storage, and signalling pathways of endocrine glands that are triggered by PPAR, AMPK, and adipokines. This method improved our comprehension of the biological mechanisms affecting adipose tissues (Ghafouri et al.). In animal food industry, the genetics and physiological mechanisms that govern skeletal muscle mass production are of prime importance to study. Satellite cells are crucial for skeletal muscle growth and regeneration. scRNA-seq was used by Lyu et al. to analyse the makeup of bovine satellite cells. According to the findings, bovine satellite cells may be divided into subpopulations with different transcriptional statuses, rates of proliferation, and myogenic potential. The current study by Lyu et al. also indicates the existence of FAP cells in bovine skeletal muscle, which may help researchers devise new methods for enhancing these features or find the DNA sequences and variants linked to them in cattle (Lyu et al.).

Lameness in cattle is frequently brought on by two non-infectious claw lesions: sole ulcers (SU) and white line disease (WLD). Sensitivity to SU and WLD is influenced by both hereditary and non-genetic variables, and protection can be accomplished by genetic methods and herd management. Genome-wide association studies (GWAS) were conducted using generalised linear mixed model (GLMM) regression, random forest (RF) and a chunk-based association testing (CBAT) technique to find susceptibility loci for WLD, SU, SU and/or WLD, and any sort of non-infectious claw damage. Potential genes for skeletal growth and mineralization, keratin, adipose tissue, wound healing and skin lesions were present in the linked areas on BTA8 and BTA13. The abundance of correlations found in this study conducted by Lai et al., as well as previous studies that showed the intricacy of the genetic background underlying non-infectious claw lesion vulnerability (Lai et al.). Lameness in an animal incurs financial burden on the animal owner, making it the third largest cause of culling an animal after mastitis and infertility. This condition is caused by foot lesions which can be either infectious or non-infectious in nature. Digital dermatitis (DD), WLD and SU are the most frequent causes. A study was performed by Lai et al. to find the loci influencing the association between the genetic causes of lameness and other prevalent health conditions. Estimates of the genetic connection between DD and SU, WLD and SU were all substantially different from zero (*p* < 0.05), whereas estimates of the genetic association between mastitis and DD, SU with metritis, and DD with milk fever were just suggestive (*p* < 0.1). These estimations of the five genetic correlations were all positive. Lai et al., observed that selection against disorders of foot may also reduce vulnerability to other health disorders because of the significant genetic association estimations between foot disorders and other illnesses (Lai et al.).

GWAS provide opportunities for comprehensive improvement of buffalo by finding genetic variants connected with lactation and reproductive features. The work conducted by Vohra et al., intends to find novel SNPs linked with milk output, lactation consistency, milk composition and fertility features at the genomic level in Murrah buffalo using the genotype-by-sequencing technique. To run GWAS on fat percentages and SNF percentages independently, more than 38,000 SNPs were employed. GWAS was also conducted on 305 days’ worth of milk output to test lactation consistency. SNPs were found to be substantially linked with the first principal component, which explained the greatest fraction of variability in milk output. Breeding effectiveness, post-partum breeding interval, and age at sexual maturity were all taken into account as fertility features. Additionally, certain putative genetic areas that may play a part in controlling milk production and fertility in Murrah were found (Vohra et al.). Singh et al., conducted a GWAS to find substantially related SNPs between proteins in milk and minerals in cattle. Identifying potential genes connected to milk minerals and milk protein percentage in Vrindavani cattle was the goal of their study. Protein percentage Ca, Cu, Zn, P and Fe were found to have a strong association with BTA 7, 2, 3, 14, and 2, respectively (Singh et al.). Further, consumers mostly judge the quality of meat based on its colour. In order to aid in pig breeding programs, Liu et al. conducted a study to identify the genes associated with meat colour in Suhuai pigs. Additionally, it calculates the genetic correlation and heredity of meat colour. (Liu et al.). Using GWAS and FST testing on Suhuai pigs, six potential genes (PIK3CG, HOMER1, VCAN, PIK3CA, FKBP1B, and FABP3) and 39 possible SNPs associated to flesh colour were found. These findings pave the way for genetic modification of pig flesh colour since they have functional implications for muscle growth, lipid binding and phosphatidylinositol phosphorylation. For sheep breeding industry, ewe productivity is considered the most important economic trait. Genes influencing fertility and lamb growth following parturition in Iranian Baluchi sheep were discovered using GWAS methods and gene set enrichment analysis (GSEA). The notable SNPs within or close to the genes RDX, ARHGAP20, FDX1, THBS1, ZC3H12C, and EPG5 were linked to composite features at birth. The identified pathways and genes have roles relevant to pregnancy, such as autophagy in the placenta, calcium ion transport, placental development and progesterone release and maternal immunological response. Genes NR2C1, HSD17B4, RSU1, VEZT, CUBN, PRLR, VIM. and FTH were discovered to be associated with composite features at weaning. According to Esmaeili-Fard et al., the findings imply that calcium ion transport throughout pregnancy and milk feeding lambs following parturition had the greatest influence on weight gain in comparison to other maternal origin effects (Esmaeili-Fard et al.). Liao et al. analysed the selectively bred group of meat rabbits and used a non-linear mixed model to fit growth curves and measure the effects of SNPs throughout the entire genome. The genetic organization of growth parameters, which is useful for applying genome selection, was also disclosed in this study, which is the first report of GWAS based on single-step NMM for longitudinal traits in rabbits. The logistic model, comprised of 87,704 SNPs in rabbits, was ideally selected and subjected to GWAS using this approach. The two growth indices mature weight (A) and maturity rate (K) were shown to be simultaneously impacted by a total of 45 important SNPs spread across 5 chromosomes. Seven positional genes were proposed as potential candidates influencing meat rabbit growth, including GBA3, KCNIP4, LDB2, PPARGC1A, GNA13, SHISA3 and FGF10 (Liao et al.).

One of the most important economic traits in swine industry which determines its profitability is the meat quality and genetics has an important role in determining such traits. Ardestani et al., conducted a study to evaluate the accuracy of standard BLUP prediction with several prominent genetic assessment techniques such as GBLUP, ssGBLUP, and BayesC for back-fat thickness (BFT), average daily gain (ADG) and loin muscle depth (LMD) parameters in Canadian swine populations. Despite the absence of any significant differences (*p* > 0.05) between the prediction accuracies produced from these genomic approaches in each scenario, ssGBLUP and BayesC techniques often demonstrated the maximum predictive performance and unbiasedness, respectively (Salek Ardestani et al.).

The milk cattle breed known as Sahiwal is indigenous to the Indian subcontinent. They are renowned for their high milk production, exceptional ability to adjust to the hot, humid conditions that prevails in their native territory, and resilience to parasites, ticks, and tropical diseases. This study uses a genotyping INDUS chip to investigate the signature of selection in the genome of Sahiwal cattle. The potential signs of selection in Sahiwal cattle were discovered in 14 important regions of the genome. The most prominent locations were mapped onto BTA 6, 20, and 23, and the *p*-values from several univariate statistics were combined into a composite signal using the DCMS methodology. The selection signatures, which include important candidate genes linked to features including coat colour, milk fat percentage, sperm membrane integrity, and carcass qualities, are present in BTA 6. There are genes related to bovine *tuberculosis* sensitivity and parasite infestation tolerance located in a significant area on BTA 23 at the 17-Mb region. Illa et al., came to the conclusion that the extremely relevant genetic areas contributed to Sahiwal becoming one of the best milk cow breeds for the tropics (Illa et al.).

Bioinformatic techniques were used to streamline and accelerate the process into a single step in order to identify the causative variants of full penetrance recessive genetic diseases using only nine whole genome sequenced animals. A novel mutation causing prenatal mortality in Irish moiled calves was found utilising whole genome sequencing of only three situations and six carriers. The variant call format (VCF) data file of these 9 animals was examined using four different techniques: autozygosity-by-difference (ABD), genotyping criteria (GCR), variant prediction scoring, and enrolled SNP information. Only one site (Chr4: g.77173487A>T (ARS-UCD1.2 (GCF 002263795.1)) out of around ten million variants in the VCF file was recognised by all these techniques. The glucokinase gene contained a splice acceptor variation at this location (GCK). This study by Pollott et al., showed that only a limited number of cases and controls are needed, and that controls should exceed cases by a ratio of 2:1 and should be less closely related to cases (Pollott et al.). The Pashmina goat genome published in the current edition by Bhat et al., may offers an opportunity to understand the biology of development of this pricy and luxurious fiber (Bhat et al.). This study is one of the first attempts at providing Pashmina genomics and transcriptomic information. Using the Illumina HiSeq 2500 sequencer, a total of 294.8 GB (>100X coverage) of the whole-genome sequence data was generated from a 26 months old male Changthangi Pashmina goat. Moreover, the genomes of a wild goat and a Pashmina goat were compared, and the results showed a total of 2,823 high impact single nucleotide variants as well as minor insertions and deletions that may be related to the evolution of Pashmina goats. The complex traits of the Pashmina goat, such as annual fibre cycling, defence mechanisms against hypoxia, a method of surviving in extremely cold temperatures, adjustment to a meagre diet, and distinctions of Pashmina fibre from other fibres to avoid marketing practises, can also be understood with the aid of this study.

The majority of the effort in genomic selection has focused on computational efficiency and prediction accuracy, but computing restrictions are becoming an increasingly critical factor that must be considered. A Bayes-based genomic selection model named FMixFN was created in a study by Xu et al. It combines consistent prediction ability and computational effectiveness. The needs of large breeding enterprises or combined breeding programs could be satisfied by the reliable, big data-oriented genomic selection approach known as FMixFN. The FMixFN technique is publicly available at https://zenodo.org/record/5560913 (Xu et al.). Vaughn et al., used a mixed linear model in fixed effects and random effects to investigate the expression of Actinin-3 (ACTN3) gene, which encodes a muscle-specific structural protein, in correlation to the feeding efficiency phenotype in *Bos taurus* - *Bos indicus* crossbred steers (Vaughn et al.). It was assumed that animals with a higher proportion of fast-twitch muscle fibres are comparatively feed inefficient because the ACTN3 is exclusive to fast-twitch muscle fibres and absent from slow-twitch muscle fibres.

The ovulation rate, a critical reproductive characteristic for goats, determines the top limit of the female’s litter size. The rate of ovulation in the ovary is influenced by follicle growth and development. To anticipate how lncRNAs and miRNAs would interact, Tao et al., sequenced and analysed the mRNA expression profiles of pre-ovulatory follicles from goats. Out of the 895 lncRNAs that were found, 88 displayed a clear difference in expression, suggesting important impacts on the ovarian follicles in goats. LncRNA XR_311113.2 might operate as a chi-miR-424-5p sponge. This research demonstrates that LncRNAs may be a valuable new area of investigation in the context of ovarian follicular development (Tao et al.). Yak, a unique reservoir of genetic material that primarily lives on the Qinghai-Tibet Plateau. A clear heterosis in production performance may be shown in cattle-yak, the hybrid offspring of yak (*Bos grunniens*) and cattle (*Bos taurus*). Through RNA sequencing, 7,126 mRNAs, 791 lncRNAs, and 1,057 circRNAs that differ in expression between yaks and cattle-yaks in the longissimus dorsi muscle were discovered. The ncRNAs were discovered to be associated with the proliferation and differentiation of myoblast cells, skeletal development, and signalling pathway of muscle growth using bioinformatics techniques. Huang et al. claim that this study can be utilised as a benchmark to establish the molecular framework for understanding muscle growth (Huang et al.). The blue egg is biologically interesting as well as economically significant for consumers, egg sellers, and scientists. Studies conducted in the past mostly focused on protein-coding genes to understand the genetic pathways behind pigment deposition. The underlying mechanism by which non-coding RNAs affect the pigment deposition in various eggshell colours is still unknown. Profiled the uterine gland transcriptome (lncRNA and mRNA) of 15 Changshun blue eggshell layers using RNA sequencing. 8 and 22 lncRNA-gene pairings were predicted by combining analyses of lncRNA and mRNA profiles. According to mRNA sequencing, the majority of pathways were mostly focused on lipid-related metabolisms. The found lncRNAs influence immunological and lipid-related metabolisms, as well as pigment disposition, to exert similar effects on colour creation (Chen et al.). The integrated co-expressed transcriptomes, i.e., mRNA and miRNA, were investigated in primary bovine myoblasts following Resveratrol treatment. According to Hao et al., this study improved our knowledge of the functions of RSV in inducing miRNA, the features of DE miRNAs in the important co-expressed component that regulate mRNAs, and it discovered new potential transcription factors and miRNAs for traits associated with meat quality (Hao et al.).

He et al., investigated the link between ACSF3 expression and cellular synthesis of triglycerides (TG) by silencing and over-expression of ACSF3. As a result of the discovery that ACSF3 regulates cytoplasmic triacylglycerol and long-chain PUFA levels, polymorphism may be used as a diagnostic biomarker for forthcoming marker-assisted selection in the production of elevated lipid accumulation traits in beef cattle (He et al.). Heat stress in cattle exhibit a direct impact on rumen health by affecting the processes of fermentation and metabolism of rumen papillae proliferating the rumen papillae. It may adversely affect the physical membrane of the rumen epithelium to a significant degree by increasing corneum loss. HS up-regulated biological processes such sister chromatid segregation while down-regulating MAPK and NF-kB cell signalling pathways, according to an examination of the rumen papillae’s transcriptome profile. These pathways were linked to DNA replication and repair, amino acid metabolism, and other pathways that were problematic. TLR4 or Tight junction protein signalling expression did not change specifically in response to heat stress, according to Guo et al., indicating that HS had a limited detrimental impact on the ruminal epithelium’s physical barrier but did not completely destroy it. The development of mitigation techniques as well as the productivity of lactation cows is both impacted by the increase in amino acid metabolism in rumen papillae (Guo et al.).

A basic description of the inheritance patterns of alternative splicing in broiler and layer chickens is provided by Qi et al. to best explain post-transcriptional regulation during hybridization. The White Leghorn and Cornish Game chicken breeds, which have markedly different body types and reproductive characteristics, were crossed in an experiment, and the muscle, brain, and liver tissues were then sequenced to determine the inheritance patterns. High tissue and strain specificity is suggested by alternative splicing profiles. The majority of the alternative splicing genes had patterns that were conserved across all three organs, according to a study between parental strains and hybrid crossings. This study gives an overview of the alternative splicing inheritance patterns in these chicken breeds (Qi et al.). Khan et al. developed the first comprehensive buffalo user-friendly web genomic resource (BuffGR). It contained genetic research on five buffalo breeds with major commercial importance: the Mediterranean, Egyptian, Bangladeshi, Jaffrarabadi, and Murrah (Khan et al.). The website’s database contains information on 4504691 SSR markers across all breeds, 1458 distinct circRNAs, 37712 lncRNAs, and 938 miRNAs from the genomic sequences of the Mediterranean breed of buffalo. This data could be utilised to research the genetic diversity of many buffalo breeds. It is also possible to research post-transcriptional regulation and its function in several bovine diseases. The BuffGR can be found at http://backlin.cabgrid.res.in/buffgr/.

Precision when population size is small is a significant barrier to their genomic selection; during the past several years, variable selection approaches with diverse variance have been proposed to increase breeding value accuracy. While these models might be more accurate than conventional and genetic forecasts, they also carry a correspondingly higher breeding value bias and dispersion. Mancin et al., used a number of diverse techniques in his study to increase the precision of genomic selection in a small population. They were chosen using a variety of algorithms, including XGBoost, penalised regression, and recursive feature eliminations (Mancin et al.). Variable selection ssGBLUP, especially XGBoost, has prediction accuracy that is higher than other ssGBLUP methods without the exaggerated bias and dispersion that come with weighted ssGBLUP. In this study, machine learning algorithms are used, which may provide a solution to the problem of genomic selection in small populations, such as the local cow population. The techniques described in his study may help preserve indigenous cow breeds, study them, and boost their economic competitiveness. For carcass evaluation in meat industry, rib eye area is an important index used. Since this indicator is inherited and possesses genetic diversity, it can be used to improve the genetic makeup of sheep. This study conducted KASPar genotyping on five SNPs, demonstrating that the rib eye region is highly linked with SNPs in LOC105611989, DPP6, and COL12A1. These SNPs could be used as genetic markers for rib eye area molecular breeding. The findings published by Zhao et al., offer genetic factors evaluated on the rib eye region and guidance for breeding Hu sheep based on carcass features (Zhao et al.).

